# Nano-Scale Stiffness and Collagen Fibril Deterioration: Probing the Cornea Following Enzymatic Degradation Using Peakforce-QNM AFM

**DOI:** 10.3390/s21051629

**Published:** 2021-02-26

**Authors:** Ahmed Kazaili, Hayder Abdul-Amir Al-Hindy, Jillian Madine, Riaz Akhtar

**Affiliations:** 1 Department of Mechanical, Materials and Aerospace Engineering, School of Engineering, University of Liverpool, Liverpool L69 3GH, UK; ahmed_mohammed381@yahoo.com; 2 Department of Biomedical Engineering, College of Engineering, University of Babylon, Babylon, Hillah 51002, Iraq; 3 College of Pharmacy, University of Babylon, Babylon, Hillah 51002, Iraq; phar.hayder.abdul@uobabylon.edu.iq; 4 Department of Biochemistry and Systems Biology, Institute of Systems, Molecular and Integrative Biology, Faculty of Health and Life Sciences, University of Liverpool, Liverpool L69 7BE, UK; j.madine@liverpool.ac.uk

**Keywords:** Peakforce-QNM, AFM, cornea, collagenase, amylase, nanomechanics, collagen, collagen fibril morphology, keratoconus, collagen fibril diameter

## Abstract

Under physiological conditions, the cornea is exposed to various enzymes, some of them have digestive actions, such as amylase and collagenase that may change the ultrastructure (collagen morphology) and sequentially change the mechanical response of the cornea and distort vision, such as in keratoconus. This study investigates the ultrastructure and nanomechanical properties of porcine cornea following incubation with α-amylase and collagenase. Atomic force microscopy (AFM) was used to capture nanoscale topographical details of stromal collagen fibrils (diameter and D-periodicity) and calculate their elastic modulus. Samples were incubated with varying concentrations of α-amylase and collagenase (crude and purified). Dimethylmethylene blue (DMMB) assay was utilised to detect depleted glycosaminoglycans (GAGs) following incubation with amylase. Collagen fibril diameters were decreased following incubation with amylase, but not D-periodicity. Elastic modulus was gradually decreased with enzyme concentration in amylase-treated samples. Elastic modulus, diameter, and D-periodicity were greatly reduced in collagenase-treated samples. The effect of crude collagenase on corneal samples was more pronounced than purified collagenase. Amylase was found to deplete GAGs from the samples. This enzymatic treatment may help in answering some questions related to keratoconus, and possibly be used to build an empirical animal model of keratoconic corneas with different progression levels.

## 1. Introduction

There is a need to investigate and develop a better understanding of corneal ultrastructure and biomechanics at the nano-level. Understanding corneal ultrastructure and its response to chemicals is important for a number of ocular disorders, such as keratoconus that is characterised by a significant deterioration in the collagenous network resulting in a cone-shape cornea.

The human cornea consists of five layers, in which the stroma represents about 90% of its thickness. The stroma is composed of many lamellae that are parallel to the corneal surface. The stroma mainly consists of Type I collagen fibrils that are arranged in a high degree of lateral order and run in the same direction as their lamellae. Collagen fibrils consist of several collagen molecules that are aligned together in staggered arrangement to form a banding pattern, which is referred to as D-periodicity. Collagen fibrils in human corneas have relatively uniform diameters of 32.5 ± 1.5 nm and a D-periodicity of 65 nm [1,2], which may be slightly higher in porcine corneas [3]. Collagen fibrils of the stroma are associated with proteoglycans that keep them aligned and give support to provide the overall shape and strength of the cornea. Proteoglycans contain chains of glycosaminoglycans (GAGs), which are polysaccharide molecules that attract water and are thought to provide the extracellular matrix with additional physical properties not provided by collagen fibrils alone [4].

The cornea is exposed to a number of enzymes that are either secreted by lacrimal gland or produced by the corneal cells, such as keratocytes. Some of these enzymes, such as amylase [5] and collagenase [6] are believed to have digestive actions in the stromal layer of the corneas. It was also found that these enzymes cause a reduction in collagenous tissue stiffness; therefore, they may contribute in the progression of keratoconus [7].

Alpha-amylase is an active enzyme in the tear fluid that is thought to increase in patients with keratoconus [8], where it was found that it can decrease corneal stiffness [9]. Previous studies have used extensometers to examine the effect of alpha-amylase on corneal sections [9,10]. They found that corneal stiffness decreases with alpha-amylase incubation. The corresponding alteration in tissue ultrastructure has not been investigated previously.

The other digestive enzyme in the cornea is collagenase, which attacks the peptide bonds of the triple helix region on collagen. It is found in epithelial and stromal cells and can be released into the stromal layer in response to trauma for biomechanical modulation of the collagenous network [6,11,12,13]. Collagenase has been approved by United States Food and Drug Administration (FDA) as an injection to improve the range of motion in joints affected by advanced Dupuytren’s disease [14]. In the eye, collagenase was found to increase in patients with keratoconus [12,15,16] due to reduction of its inhibitor [17,18]. However, no ultrastructural or nanomechanical investigation has been conducted to show its activity on corneal tissues.

The investigation of ultrastructural topography and biomechanical properties of the cornea requires a technique that is less destructive to the samples, such as the atomic force microscope (AFM). Other techniques such as scanning electron microscopy (SEM) and transmission electron microscopy (TEM) may provide ultrastructural details of the cornea, but samples need to be sputter-coated and dried extensively, affecting measurement of diameters and axial D-periodicity of collagen fibrils [19] as they are altered due to significant dehydration [20]. Therefore, AFM has been selected in this paper to capture in vitro ultrastructural topography and nanomechanical properties without extensive dehydration and sputter-coating which leads to alteration of the ultrastructure through extensive dehydration [3,19,20].

The use of AFM in the investigation of corneal diseases can help in addressing many questions. Early AFM studies focused on the collagen fibrils characterisation in mammalian corneas [21]. Other studies yielded elastic properties of corneal layers [22,23] which is useful for understanding mechanical properties in healthy and diseased corneas. For example, AFM was used to analyse photoablated stromal corneas in comparison to untreated samples [24]. The study showed undulations and granule-like features on the ablated stromal surface when a 193 nm excimer laser was used, and confirmed the precision of laser surgery in removing submicrometric amounts of the stroma. AFM was also utilised to investigate the ultrastructural topography in stromal layer of human corneas following collagen cross-linking treatment with riboflavin and ultraviolet-A light [25]. However, to date, AFM has not been used to examine ultrastructural changes in either keratoconic corneas or corneas exposed to enzymatic degradation in vitro. 

This study takes advantage of recent advances in AFM such as PFQNM-AFM (Peak Force Quantitative Nanomechanical atomic force microscopy), which enables fast acquisition and mapping of a sample’s mechanical properties [26]. PFQNM-AFM has demonstrated utility for characterising localised mechanical properties of collagen-rich tissues such as the sclera [27] and arterial tissue [28]. Rapid acquisition of tissue ultrastructure is achievable with comparable high-resolution mechanical property maps.

With PFQNM-AFM, in this study, we investigate ultrastructural and nanomechanical changes following enzymatic incubation of porcine corneal tissue with amylase and collagenase. We assess GAG depletion following amylase incubation, and also assess the difference between crude collagenase and purified collagenase on corneal degradation. We exploit the ability of the AFM microcantilever to act as a “force sensor” at the nano-scale for this novel application to characterise mechanical degradation in the cornea following in vitro degradation.

## 2. Materials and Methods

### 2.1. Tissue Samples

Eighteen porcine eyes were sourced from 5 to 6 month old pigs from a local abattoir shortly after slaughter. They were divided into three main groups (6 corneas each group); amylase-group, crude collagenase group, and purified collagenase group. The corneas were dissected immediately on arrival (within an hour from slaughter) at the University of Liverpool. Corneal samples were chosen from the apex (3 mm diameter) after desquamating the epithelial layer using a cotton-tipped applicator and tweezers. The preparation procedure included snap freezing and cryosectioning to make the corneal sample ready for AFM experiments.

After dissecting, the samples were carefully rinsed with Phosphate Buffered Saline (PBS) (Sigma-Aldrich, Dorset, UK), placed in a cryomould with appropriate orientation and embedded in the non-infiltrating optimum cutting temperature resin (Tissue-Tek, CellPath, Powys, UK).

Cryosectioning was performed by utilising a Leica cryostat (model CM1850, Leica Microsystems Ltd., Milton Keynes, UK) to section the frozen corneal samples to a thickness of 5 µm. In the amylase group (6 corneas), eleven sections of 5 µm thickness were taken from the first third of each cornea. The first third was defined as the anterior stromal layer of 150 µm after the epithelium. In the crude collagenase group (6 corneas), five sections of 5 µm thickness were taken from the first third of each cornea. A similar number of sections were prepared from the purified collagenase group. In all corneas, sections were taken after a depth of 20 µm from the anterior surface of the stroma. Sections were then stored at −80 °C until they were tested.

### 2.2. Enzymatic Treatment

For the amylase group, the cryosectioned tissue (n = 66) was treated with α-amylase (type Aspergillus oryzae, Sigma-Aldrich, Dorset, UK) for 40 min. Amylase was diluted in PBS to varying concentrations (0.2, 0.4, 0.6, 0.8, 1, 1.2, 1.4, 1.6, 1.8, and 2 mg/mL). The control sections in this group (n = 6) were incubated in PBS only, for the same period. One drop (40 mL) of amylase at 37.5 °C was applied on the amylase treated group and kept at 37.5 ± 1.1 °C for 40 min. Afterwards, the corneal sections were washed with cold PBS (4 °C) and left in air for 18 min in preparation for AFM testing.

For the crude collagenase group, the samples (n = 30) were incubated with crude collagenase (type *Clostridium histolyticum*, Sigma-Aldrich, Dorset, UK) for 15 min at 37.4 ± 1.8 °C. The treated samples of this group (n = 24) were incubated with one drop (40 mL) of crude collagenase (0.05, 0.1, 0.15, and 0.2 mg/mL) at 37.5 °C that were diluted in PBS. Those samples were snap-washed firstly by a cold (4 °C) aqueous solution of dichloromethylene diphosphonic acid disodium (NaDTA) at a concentration of 1 mg/mL to inhibit the activity of the collagenase; and subsequently by PBS twice and tested after 18 min. The control samples of this group (n = 6) were incubated in PBS for 15 min and snap-washed with NaDTA and PBS.

For the purified collagenase group, the samples (n = 30) were treated with varying concentrations of purified collagenase (type *Clostridium histolyticum*, Sigma-Aldrich, Dorset, UK) at 37.5 °C (0.05, 0.1, and 0.15 mg/mL) for 15 min. Six samples served as controls, which were incubated in PBS only, for 15 min to investigate the effect of snap-washing the sections with cold (4 °C) NaDTA. The treated samples (n = 24) of this group were also snap-washed with cold NaDTA and PBS following the enzymatic incubation and tested after 18 min. Table 1 summarises these groups and treatment solutions.

### 2.3. PFQNM-AFM Method

A Bruker MultiMode 8 AFM with E-piezoelectric scanner (Bruker Nano Inc., Nano Surfaces Division, Tucson, AZ, USA) was utilised to investigate the topographical and elastic property changes of the cryosectioned samples following enzymatic treatment. The PFQNM mode in air was used, which is most suited for biological samples with structural heterogeneity [29,30]. This mode is characterised by its ability to control the applied forces to the sample (or the peak force), which allows indentations to be limited to several nanometres that both maintains resolution and prevents sample damage. In addition, Peak Force QNM mode allows measurements at an extremely wide range of elastic moduli (1 MPa to 50 GPa) [30]. This mode uses the Derjaguin–Muller–Toporov (DMT) model and a curve fitting process of the unloading portion of the force to calculate elastic modulus, as outlined in other papers [30,31].

The AFM experiments were conducted with a silicon probe with a rectangular tip, type RTESPA-300 (Bruker Nano Inc., CA, USA). It was used due to its capabilities of capturing high resolution topographical images and its ability to measure a wide elastic modulus range of the samples being tested (200 MPa–5000 MPa). The nominal tip radius was 10 nm, and spring constant of the cantilever was 22 N/m with a resonance frequency of 300 kHz.

Relative calibration of the AFM was performed before every test. The calibration procedure was used to define the parameters of the PF-QNM mode. These parameters were measured relative to a known reference sample, Vishay Photostress PS1 Polymer (Vishay; Wendell, NC, USA). This reference sample had a known elastic modulus of 2.7 ± 0.1 GPa, which was utilised to calibrate the AFM and estimate the tip radius. A direct method of thermal tuning was carried out to measure the spring constant of the cantilever. Finally, deflection sensitivity was calibrated to convert Volts measured on the photodetector to nanometres of motion, which was performed by measuring a force curve on an “infinitely stiff” surface relative to the chosen cantilever. Therefore, a sapphire sample (Sapphire-12M; Bruker Nano Inc., Nano Surfaces Division, CA, USA) was utilised, which is stiff enough that the cantilever does not indent it during the force curve measurement.

With the use of the optical microscope integrated with the AFM, images of the cryosectioned corneas were captured for 3 different locations on each sample. Topographical images were collected at 5 × 5 µm and also 1 × 1 µm. The 1 × 1 µm images were suitable to visualise individual collagen fibrils. Peak force error images, which are referred to as the derivative of topography image [32], were also captured to visualise collagen fibrils orientation. The peak force frequency and amplitude were set to 2 kHz and 150 nm respectively. A scan rate of 0.799 Hz was utilised for all samples. The 1 × 1 µm images were captured with 256 horizontal lines, and each line was scanned at a resolution of 256 pixel/line. The 5 × 5 µm images were scanned with 512 horizontal lines, and each line was scanned at a resolution of 512 pixel/line. These setting were chosen following many trials to obtain good quality images whilst minimising artefacts. In addition, these settings were recommended by the manufacturer for biological tissues for optimum results.

### 2.4. Collagen Fibril Analysis

**Figure 1:**
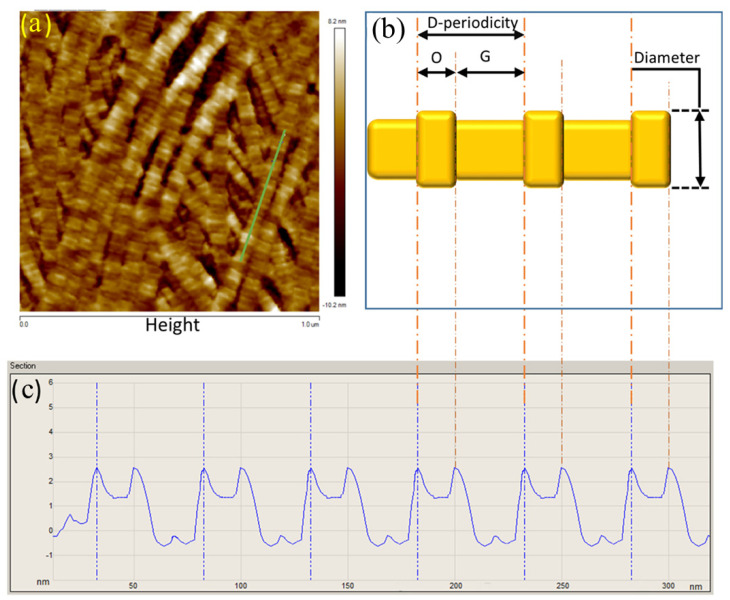
Collagen fibril surface profile. (**a**) Height image of the anterior lamella of a porcine cornea obtained with Atomic force microscopy (AFM). The green line in image (**a**) shows the area selected for analysis. (**b**) Schematic diagram showing collagen fibril morphology. "O" and "G" refer to overlap and gap zones. (**c**). Line profile generated from the green line shown in (**a**) with the corresponding O, G and periodicity shown with reference to (**b**). The peak-to-peak distance was measured manually to, which represents the D-periodicity. This analysis process was carried out 3 times as a minimum to calculate the average value of collagen fibril diameter and D-periodicity for each image. For this image, collagen fibril diameter and D-periodicity were 55.5 ± 2.4 nm and 67.8 ± 1.1 nm, respectively.

Collagen fibril diameter and D-periodicity were measured with NanoScope Analysis 1.7 software (Bruker Nano Inc., Nano Surfaces Division, CA, USA). Collagen fibrils that were straight with high contrast and at approximately zero inclination angle were manually selected from each height image to measure their diameter and D-periodicity. The recorded values then were averaged for each image. Figure 1 shows an example of the collagen fibril surface profile that was collected.

### 2.5. Glycosaminoglycans Quantification

Eight fresh porcine corneas were utilised for these experiments. The epithelium of the corneas was removed. Four portions of 6.5 mg in weight were cut from the anterior central region of the stroma of each cornea and organised into four groups: tissue culture (TC) group (n = 8), control group (n = 8) and amylase groups (n = 16) that was subdivided into two subgroups. Each sample of the first amylase subgroup (n = 8) were incubated with 1 mL of α-amylase (2 mg/mL) for 60 min. Samples in the second amylase subgroup (n = 8) were incubated with the same amount and concentration of the α-amylase for 120 min. Control samples were incubated with 1 mL of PBS for 120 min. TC group samples were incubated with 1 mL of TC (CARRY-C, Alchimia, Italy) for the same period. All samples were slightly shaken and incubated at 37.5 °C. These treatment solutions were assessed with the Dimethylmethylene Blue (DMMB) assay to quantify the depleted proteoglycans in the solutions [33].

### 2.6. Data analysis and Statistics

All statistical analysis were performed using OriginPro 2016 version 9.3 (OriginLab, Northampton, MA, USA). Data are expressed as mean values and standard deviations (mean ± standard deviation) unless otherwise stated. The two-sample t-test was used to test the statistical difference, with the significance level (α) set as 0.05 for all tests.

## 3. Results

### 3.1. Amylase Group

**Figure 2:**
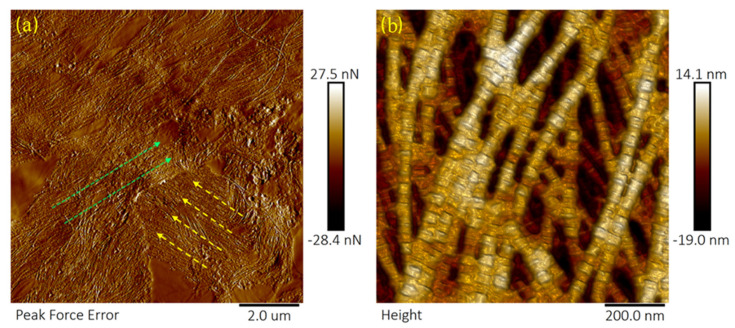
Typical topography (height) and peak force error images showing collagen fibrils of the anterior lamella of a porcine cornea (control samples). (**a**) A peak force error image of a control sample with a scan size of 10 µm. The yellow arrows show the direction of a lamella that runs at a right angle to another bundle of collagen fibrils (the green arrows). In each bundle, collagen fibrils are packed together (**b**) A height image of collagen fibrils of a control sample. Scan size was 1 µm. “Height” represents the type of AFM image. A "3D effect" filter was applied for enhancement.

Figure 2 shows representative topographical images of control samples of the amylase group. In control samples, collagen fibrils appeared to be aligned and packed together. Collagen fibrils had diameters of 55.5 ± 2.4 nm and axial D-periodicity of 67.46 ± 2.3 nm.

**Figure 3:**
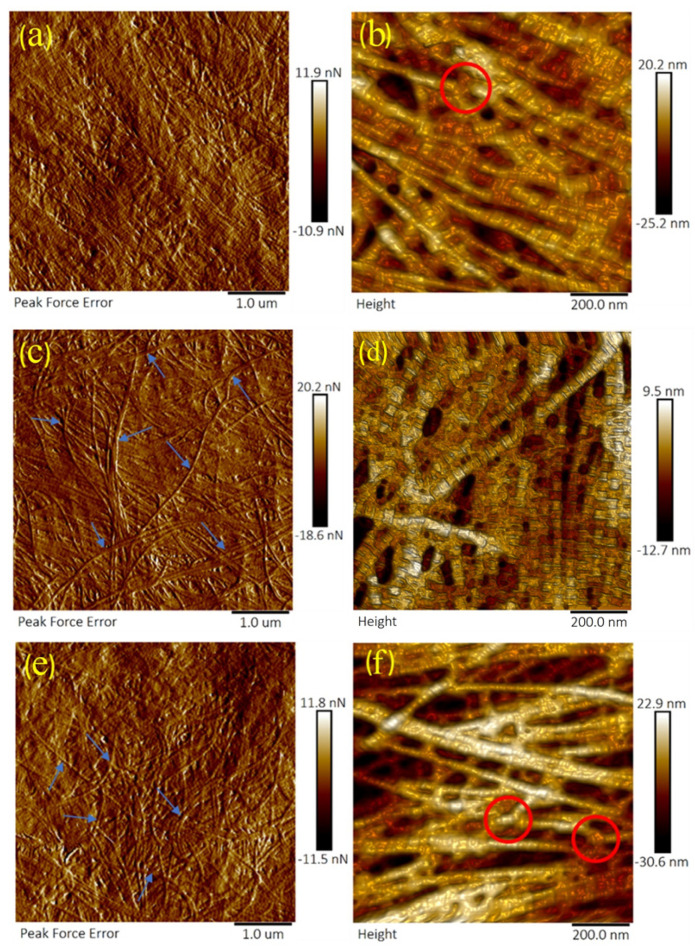
Topography and peak force error images showing collagen fibrils of the anterior lamella of porcine corneas following incubation with varying concentration of amylase. The arrows mark collagen fibrils that ran in irregular directions to other collagen fibrils following amylase incubation. Circles mark the places where the collagen fibril underwent fusion and splitting up. Sections (**a**,**b**) were incubated with 0.2 mg/mL amylase. The samples in (**c**,**d**) were incubated with 1 mg/mL amylase. Samples in (**e**,**f**) were incubated with 2 mg/mL amylase. The scan size for (**a**,**c**,**e**) was 5 µm. The scan size for (**b**,**d**,**f**) was 1 µm. A "3D effect" filter was applied for enhancement on the height images.

AFM images at different amylase concentrations are shown in Figure 3. It was also noticed in amylase treated samples that collagen fibril "splitting up" or fusion was observed. The alignment and regularity of collagen fibrils in the same lamella gradually decreased with increased amylase concentrations, see Figure 3a,c,e. Some collagen fibrils seemed to fuse with another adjacent fibril, as shown in Figure 3b,f.

**Figure 4:**
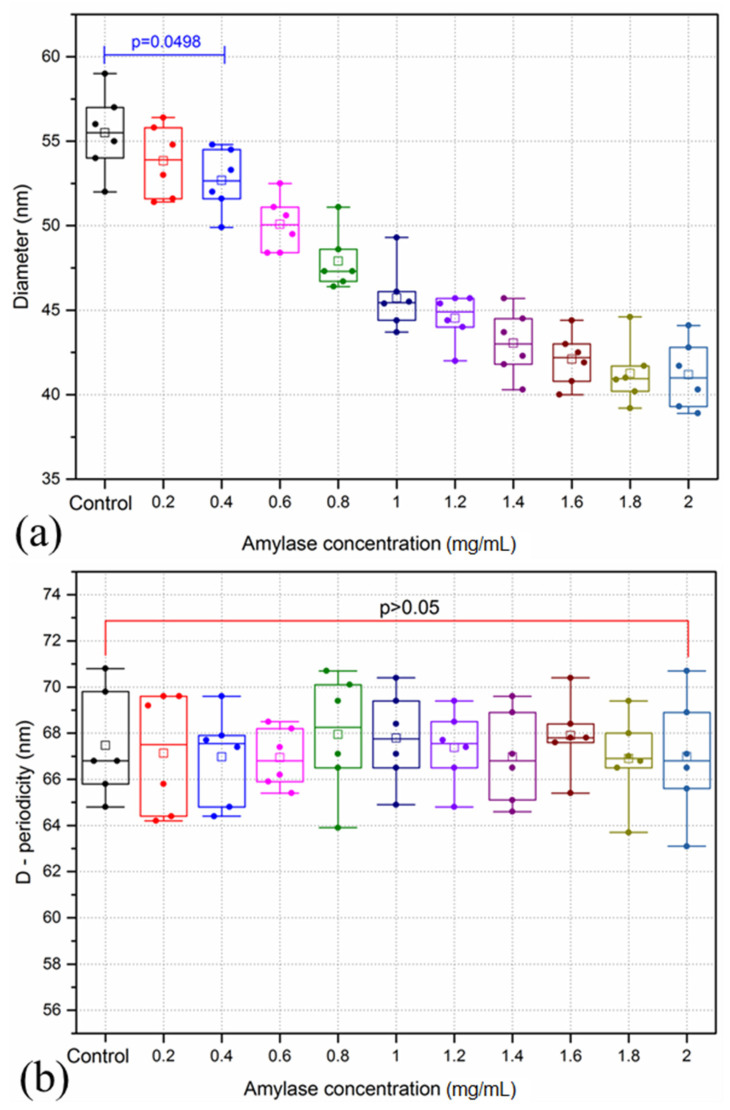
Collagen fibril diameters (**a**) and D-periodicity (**b**) of the anterior lamella of porcine cornea samples following incubation with varying concentrations of amylase. All data is represented as box plots and data overlaid with lower and upper borders of the box to represent the lower and upper quartiles, and the middle horizontal line to represent the median. The upper and lower whiskers represent 5th and 95th percentile of the data. n = 6 porcine eyes/group.

Amylase treatment was associated with collagen fibril dimeter reduction, which was significant with high amylase concentrations (*p* < 0.0498), as shown in Figure 4a. The maximum reduction in collagen fibril diameter was found in samples treated with 2 mg/mL amylase concentration, with a significant reduction of approximately 26% relative to the control group (*p* < 0.0001). No changes in D-periodicity were observed following incubation with varying concentrations of amylase, (*p* > 0.05), Figure 4b. The maximum and minimum values of D-periodicity of amylase treated samples were 70.8 nm and 63.1 nm, respectively.

**Figure 5:**
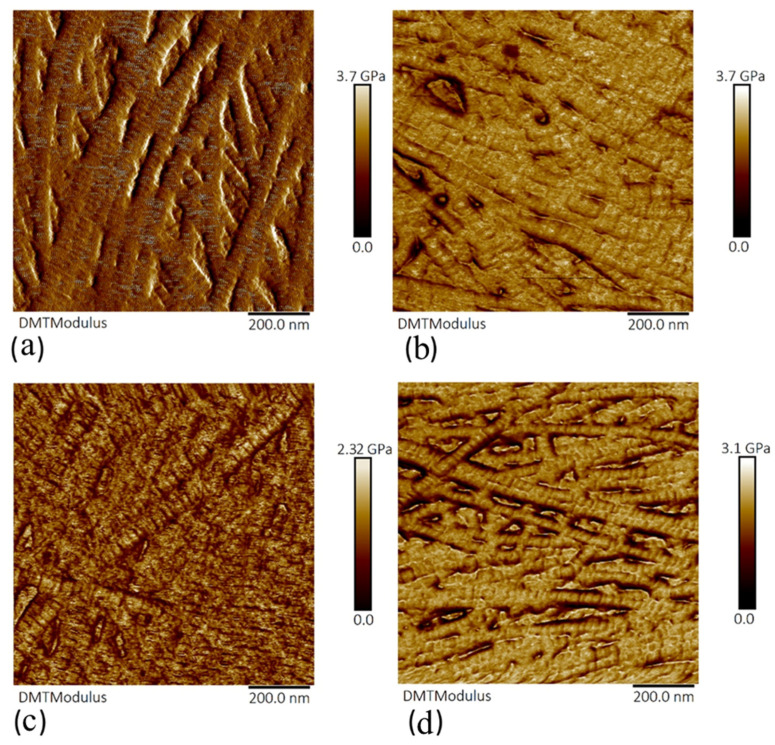
Elastic modulus maps of the anterior lamella of porcine corneas of control and amylase-treated samples. (**a**) Control sample. Amylase-treated samples incubated with (**b**) 0.2 mg/mL, (**c**) 1 mg/mL, and (**d**) 2 mg/mL. A scan size of 1 μm was selected for each image.

The elastic modulus (E) was found to decrease with amylase incubation. Example E maps following amylase treatment are shown in Figure 5. For the control samples, a mean E of 2.27 ± 0.15 GPa was recorded. E of treated corneas was decreased by 2.2% with incubation in 0.2 mg/mL amylase solution. The greatest reduction in elastic modulus of 50.2% was following incubation in 2 mg/mL amylase solution (*p* < 0.0001).

**Figure 6:**
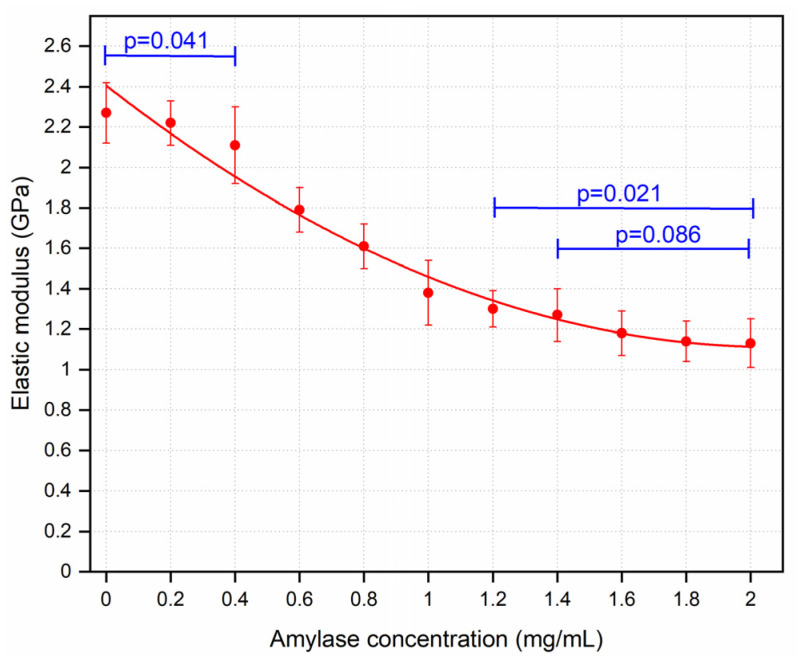
Mean values of elastic modulus for the corneal samples treated with varying concentrations of amylase. The curve was fit with a second order equation. Zero amylase concentration refers to control samples. n = 6 porcine eyes/group. Error bars represent the standard deviation.

Figure 6 shows the trend with amylase concentration. E was decreased in a negative logarithmic relation with increasing amylase concentration. However, no significance differences were detected in reduction of E when the corneas were incubated with amylase of 1.8 and 2.0 mg/mL (*p* > 0.086).

### 3.2. Crude and Purified Collagenase Groups

**Figure 7:**
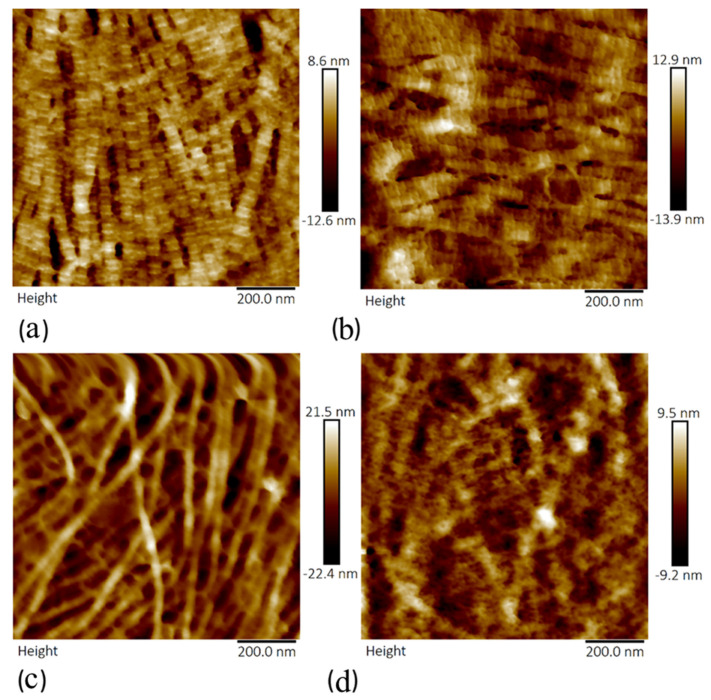
Topography images showing collagen fibrils of anterior lamella of porcine corneas following incubation with varying concentration of crude collagenase: (**a**) 0.05 mg/mL, (**b**) 0.1 mg/mL, (**c**) 0.15 mg/mL, and (**d**) 0.2 mg/mL. The scan size was 1 µm for each image.

**Figure 8:**
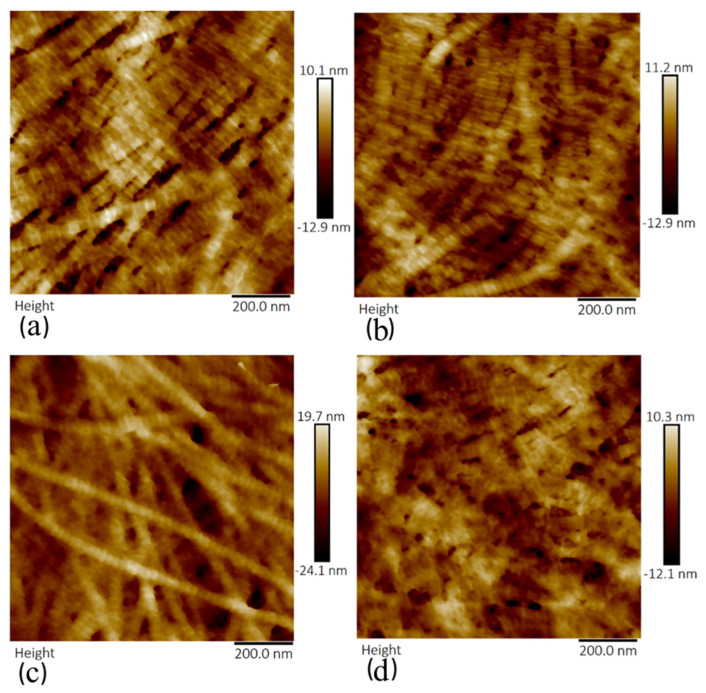
Topography images showing collagen fibrils of anterior lamella of porcine corneas following incubation with varying concentration of purified collagenase: (**a**) 0.05 mg/mL, (**b**) 0.1 mg/mL, (**c**) 0.15 mg/mL, and (**d**) 0.2 mg/mL. The scan size was 1 µm for each image.

Topographical images of sections treated with crude (Figure 7) and purified (Figure 8) collagenase revealed a significant deterioration of collagen fibrils with increasing concentration of the enzymes from 0.05 to 0.2 mg/mL. Topographical details (collagen fibril diameter and D-periodicity) were not easily identified in sections that were incubated with crude and purified collagenases of concentration 0.2 mg/mL, as shown in Figure 7d and Figure 8d. The sections treated with crude collagenase (0.2 mg/mL) showed traces of degraded collagen fibrils; however, it was possible to identify non-degraded collagen fibrils in samples that were treated with the lower concentrations of the enzymes.

**Figure 9:**
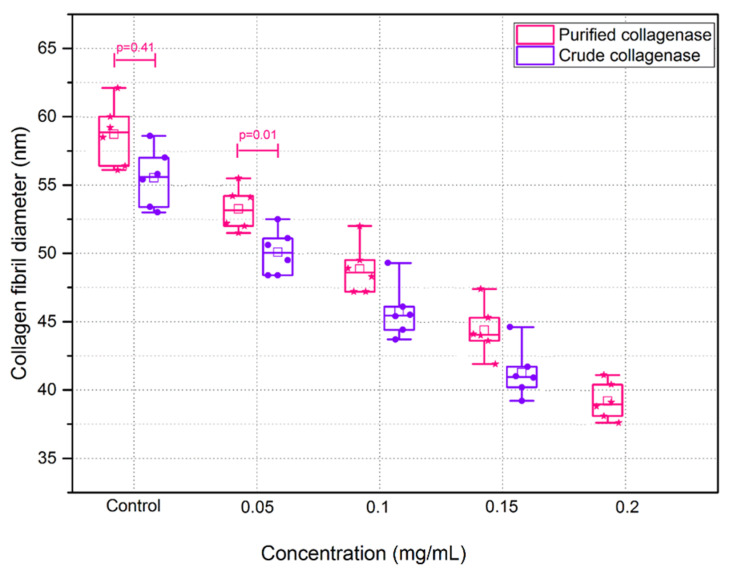
Collagen fibril diameter following incubation with varying concentrations of crude and purified collagenase (n = 6 porcine eyes/group). All data are represented as box plots and data overlaid with lower and upper borders of the box to represent the lower and upper quartiles, and the middle horizontal line to represent the median. The upper and lower whiskers represent 5th and 95th percentile of the data.

A significant reduction in collagen fibril diameter was found with both crude and purified collagenase treatment (Figure 9). The reduction in collagen fibril diameter was higher with increased concentrations of the enzymes (*p* < 0.0001). The mean collagen fibril diameter for the control samples for the crude collagenase group was 57.63 ± 2.12 nm and for the controls in the purified collagenase group, the mean collagen fibril diameter was 58.71 ± 2.26 nm. No significant difference was found between the control sections of both groups (*p* = 0.41). The minimum collagen fibril diameter was 39.16 ± 2.1 nm, observed in purified collagenase treated sections of 0.2 mg/mL, whilst no collagen fibrils were clearly visible in tissue sections where the highest crude collagenase was used.

**Figure 10:**
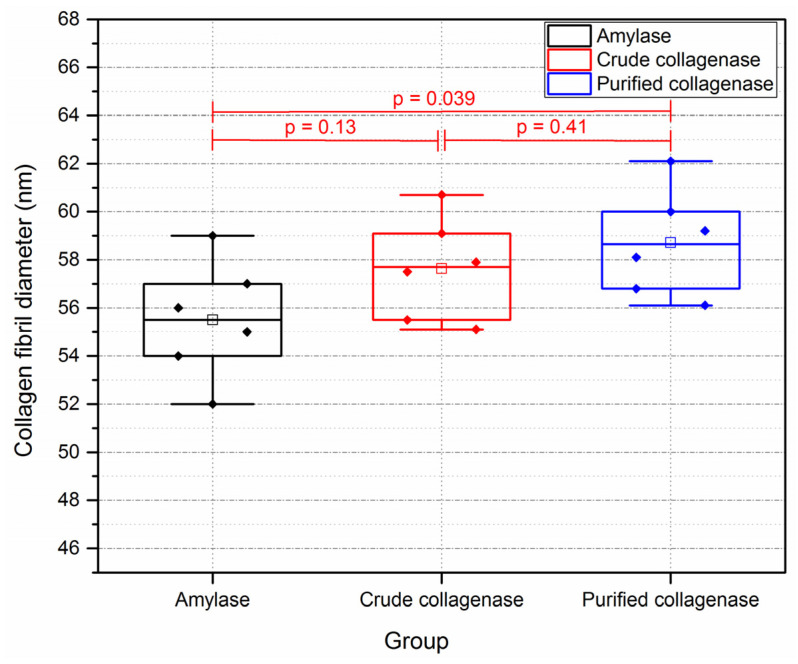
Collagen fibril diameters of control sections following incubation with PBS only for 15 and 40 min. (n = 6 porcine eyes/group.) Control sections of amylase group were incubated with PBS for 40 min. Controls in the crude and purified collagenase groups were incubated with PBS for 15 min.

The results also showed that PBS slightly decreased collagen fibril diameter. Figure 10 shows that the sections that were incubated in PBS (control sections for 40 min) had collagen fibril diameters (55.5 ± 2.4 nm) significantly less than those tissue sections that were incubated for 15 min in PBS (control sections of crude and purified collagenase groups).

**Figure 11:**
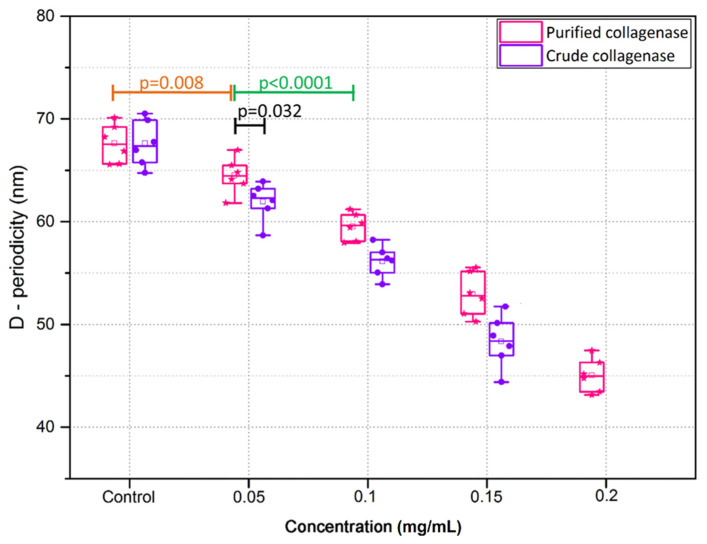
D-periodicity of collagen fibrils of porcine cornea sections following incubation with varying concentrations of crude and purified collagenase (n = 6 porcine eyes/group).

Collagen fibril D-periodicity was significantly reduced following incubation with crude and purified collagenases (*p* = 0.03) (Figure 11). No significant difference was found in D-periodicity of collagen fibrils in the control groups for crude and purified collagenase, 67.6 ± 2.3 nm and 67.4 ± 2.6 nm, respectively. The reduction in D-periodicity was approximately 8.2% in 0.05 mg/mL crude collagenase group as compared to the control sections (*p* < 0.0001), whilst at the same concentration for the purified collagenase group, the D-periodicity was decreased by approximately 4.2% in contrast to the controls. The D-periodicity was significantly decreased with increased concentration of the collagenases (*p* < 0.0001), where it was reduced to 45.1 ± 2.4 nm (approximately 33.1% lower relative to the control) at the highest concentration of purified collagenase. It was not possible to measure D-periodicity of collagen fibrils in samples that were treated with 0.2 mg/mL crude collagenase because individual collagen fibrils could not be identified.

**Figure 12:**
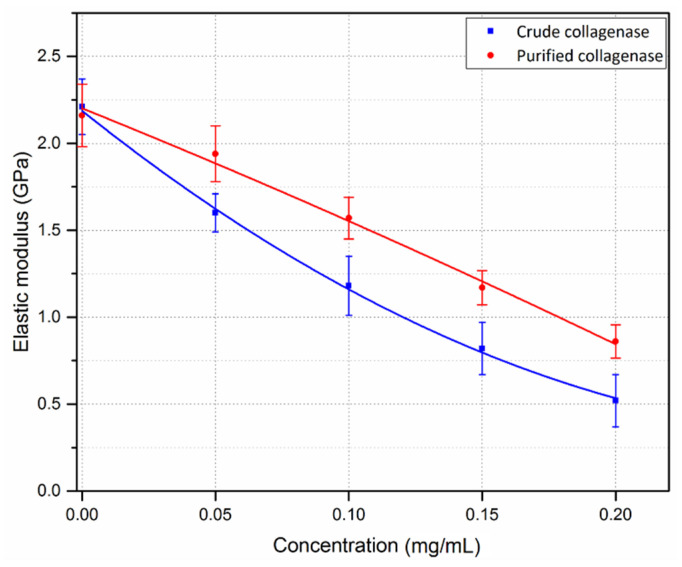
Mean values of elastic modulus of corneal samples treated with varying concentrations of crude and purified collagenase. Zero concentration refers to the control samples (n = 6 porcine eyes/group). Error bars represent the standard deviation.

The elastic modulus significantly decreased in sections that were incubated with crude collagenase for 15 min, and it decreased more with increasing concentration (*p* < 0.001), as shown in Figure 12. For the control samples, the mean E was 2.21 ± 0.16 GPa, with a 27.6% decrease following incubation with 0.05 mg/mL crude collagenase (*p* < 0.001). The maximum reduction of the elastic modulus was 76.5%, which was observed following incubation with 0.2 mg/mL crude collagenase. Similar trends in terms of E were found with the purified collagenase-treated corneas, with a significant decrease following incubation with varying concentrations of the enzyme (*p* < 0.05), Figure 12. For the controls, mean E was 2.16 ± 0.18 GPa. E appeared to decrease linearly with an increase in purified collagenase concentration. A greater reduction in E was found in the crude collagenase group as compared to the purified collagenase group. This difference was statistically significant at each concentration (*p* < 0.001). No significant difference was found in E for the controls when comparing the crude and purified collagenase groups (*p* = 0.92).

### 3.3. GAG Quantification

**Figure 13:**
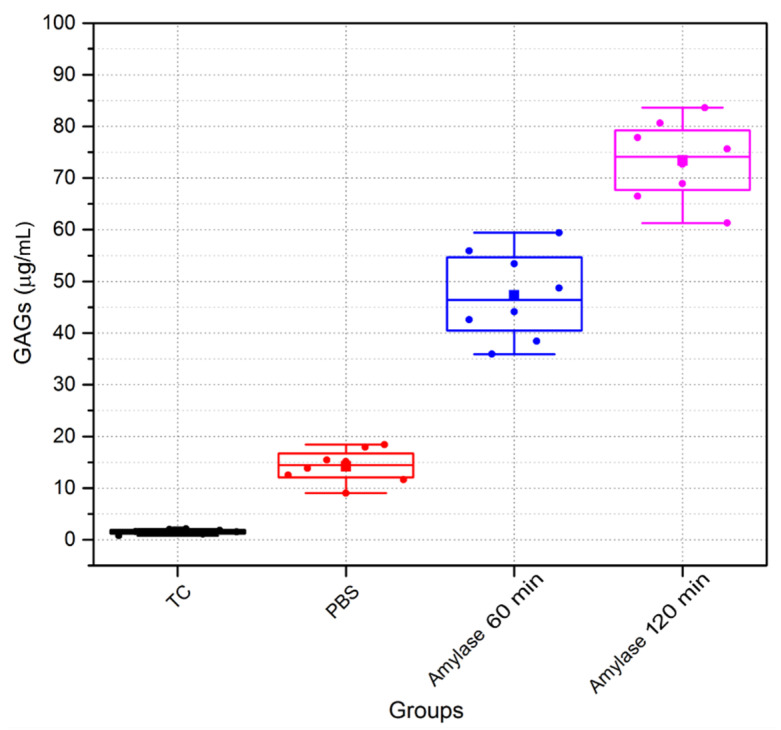
Depleted sulphated glycosaminoglycans (GAGs) of porcine corneal samples following incubation with tissue culture (TC), Phosphate Buffered Saline (PBS), and amylase. (n = 8 porcine eyes/group).

Figure 13 shows the quantities of GAGs in treatment solutions. The quantities of GAGs released in treatment solutions after 60 min and 120 min in amylase were 47.3 ± 8.4 µg/mL and 73.3 ± 7.5 µg/mL, respectively. GAG quantities released in treatment solutions of the amylase group were significantly higher than in other groups (control and tissue culture groups), *p* < 0.0001. A small quantity of GAGs (14.2 ± 3.1 µg/mL) were released in treatment solutions of the PBS group. There was little or no GAGs released in the TC group. Statistically significant differences were found among the groups (*p* < 0.01).

## 4. Discussion

This paper utilises the PFQNM-AFM mode to investigate nano-scale alterations in the mechanical properties and collagen fibril ultrastructure of the porcine cornea following in vitro enzymatic degradation. The paper presents a novel application of this fast data acquisition AFM mode, utilising the AFM cantilever as a sensor for detecting alterations in the cornea following enzymatic degradation with amylase, crude collagenase, and purified collagenase.

The use of AFM has significant advantages in investigating nanomechanical and ultrastructural details over other nano-imaging techniques (such as SEM) and over conventional mechanical testing techniques such as extensometry [34]. AFM has made it possible to observe differences in stiffness of a composite material (soft tissues) and visually distinguish between hard and soft regions. With regard to imaging, collagen fibrils can be visualised without the need for special treatments (such as metal/carbon coating) that would irreversibly alter or damage the samples [34]. Clearly, an advantage of AFM is the ability to not only record ultrastructure but also to quantify the nanomechanical properties of samples [19,35]. With the use of PFQNM mode, AFM provides a link between samples topography and its mechanical properties in nanoscale, with rapid acquisition of high-resolution mechanical property maps [30].

### 4.1. Corneal Degradation with Amylase

Collagen fibril diameters of the corneal sections were decreased following incubation with amylase. In an earlier AFM study, it was found that the collagen fibril diameter in the porcine corneal stroma is 55.6 ± 5.2 nm for hydrated sections [3], which is consistent with collagen fibril diameters of the air-dried control sections in the current study. The reduction of collagen fibril diameters following incubation with amylase suggests the important role of proteoglycans in maintaining the collagen fibril diameter. It was found that the sclera (white of the eye) of highly myopic human eyes is associated with reduction of proteoglycans and contained an increased number of smaller diameter collagen fibrils in comparison with normal human sclera, which leads to an increase in sclera elasticity [36]. That finding might justify the currently presented change in collagen fibril diameters following the proposed depletion of proteoglycans with amylase.

We found that the axial collagen fibril D-periodicity did not change following treatment with amylase. The axial periodicity of Type I collagen fibrils in normal human corneal stroma has been reported as 65 nm, with X-ray diffraction [37] and 67 nm with AFM [20], which is close to the mean values of collagen fibril D-periodicity in the control samples in this study. Given that we found non-significant changes in collagen fibril D-periodicity in the amylase treated samples, our findings suggest that collagen fibrils were not digested by the amylase.

Loss of collagen fibril orientation, splitting-up and fusion of collagen fibrils was observed following enzymatic treatment with amylase. A similar finding was also reported in sclera samples following incubation with amylase [38], suggesting that depletion of proteoglycans which are located between collagen fibrils results in this alteration to collagen fibrils.

GAGs were depleted from the stroma of the cornea samples following incubation with α-amylase. GAG depletion appears to weaken the collagenous network of the tissue. The quantity of depleted GAGs from the samples was a function of incubation time. A small quantity of GAGs was released after incubation with PBS, which could have occurred as a result of tissue swelling which may damage the tissue surface and thereby lead to GAG release.

The elastic modulus was significantly decreased in amylase treated sections. Proteoglycans act as cross-links between the collagen fibrils, which together lead to the mechanical properties of the tissue. By analogy, the depletion or break down of these cross-links leads to deterioration of the normal ultrastructural organisation of the tissue, and subsequently reduction of tissue stiffness [39]. The reduction of stiffness in collagenous tissue following amylase incubation was also stated in a number of studies [9,10,40], where amylase was utilised to deplete proteoglycans of collagenous tissues.

We found that as the concentration of amylase increased, there was a greater reduction in E, which implies that high concentrations of amylase increases the number of cleavages on α-1,4 glycosidic bonds. These bonds link the GAGs to the core protein of the proteoglycans [41]. GAGs fill the space between the collagen fibrillar network in the extracellular matrix. Depletion of these GAGs with amylase appears to disrupt the organised network of collagen fibrils, leading to a weakening of the physical properties of the collagenous tissue, as has been suggested previously [4,39].

### 4.2. Corneal Degradation with Collagenase

Both crude and purified collagenase significantly degraded corneal samples, with significant changes in the ultrastructure which were manifested by alterations in collagen fibril diameters and their structural organisation. Collagen fibrils in the collagenase treated samples (crude and purified) exhibited a reduction in diameter with increased enzyme concentration. The reduction in diameter is mainly attributed to digestion of collagen fibrils by collagenases as is the established function of this enzyme [42,43,44,45]. The current finding agrees with a previous study that used AFM to measure the adhesion force and ultrastructure of the collagen fibrils on the Achilles tendons of rats following injection of collagenase [46].

It was found that collagen fibril D-periodicity significantly decreased following incubation with collagenases (crude and purified). Hence, significant digestion of the collagen fibrils occurred with these enzymes. In support of the current results, Lee and colleagues (2011) found that D-periodicity of collagen fibrils in the Achilles tendon of rats was significantly reduced after collagenase incubation [46]. Therefore, it can be said the collagenase did not fragment the collagen fibrils but instead digested them along the fibril axis. This suggestion has been supported by previous studies, where it was reported the collagenase did result in fibril fragmentation but decreased their diameter and D-periodicity [46,47].

Collagenase decreased the stiffness of the corneal tissue, and this reduction increased with enzyme concentration. These results agree with previous studies in which bacterial collagenase reduced tissue stiffness [47,48,49]. It is well known that collagen fibrils provide structural integrity to tissues and their deterioration causes a reduction in stiffness and subsequent geometrical modifications in the tissue [37,50,51]. This deterioration can be interpreted as a reduction in collagen fibril diameters, which was found to be correlated with their stiffness [27].

Interestingly, we found that crude collagenase has a stronger effect on ultrastructural details and mechanical property deterioration than purified collagenase. This result agrees with Gaul et al. (2018) who found that arterial tissue incubated with crude collagenase showed more degradation responses to strain than those incubated with purified collagenase [42]. This is attributed to contamination of crude collagenase with other proteolytic enzymes [43]. Therefore, the ultrastructural details were not possible to be observed in samples where high concentration of crude collagenase was used.

Elastic modulus values of porcine corneas, reported in previous studies [52,53], is less than those measured by AFM (nano-scale). E of control corneas (whole thickness) tested by nanoindentation with a 100 μm flat punch [52] and inflation testing [53] were in the range of 40 to 700 kPa depending on testing conditions such as internal pressure and hydration. However, E measured on corneal sections (5 µm thick) in this study were in the range of 2 to 2.45 GPa. The difference between the testing length scale is one of the reasons for higher E measured by AFM, where elastic modulus at the macroscale reflects the bulk elastic response of the corneas in the tangential direction. E measured by AFM reflects the mechanical properties of the tissue ultrastructure. It has been reported that differences in testing scales from macro- to the micro-scale leads to higher E, which can be attributed to the tissue components being probed at each length scale [54,55]. Another reason for high values is due to lack of hydration. The tissue sections were air-dried to obtain higher resolution images of the collagen fibrils than is possible under liquid. Sample thickness also affects E where the thicker the sample, the less effect there is of the substrate on sample stiffness [56]. However, increasing the thickness of the AFM sections reduces the quality of the topographical images. Therefore, an optimal tissue thickness was required to balance image quality and reduce substrate effects.

## 5. Limitations

It was hypothesised that the thickness of the tissue sections after the enzymatic treatment would decrease however, it was not possible to measure cryosection thickness following enzymatic degradation. Reduction in tissue thickness may have impacted E. Another limitation includes testing the samples in air that resulted in elevated values of corneal elastic modulus.

## 6. Conclusions

PFQNM-AFM served as a suitable method for examining nanomechanical and ultrastructural changes in the cornea following incubation with amylase and collagenase. Amylase treatment reduces collagen fibril diameter and corneal stiffness, but not D-periodicity of collagen fibrils. The reduction in corneal stiffness and collagen fibril diameter increased with amylase concentration due to GAG depletion, which is thought to break-down proteoglycan linkages with collagen fibrils that leads to deterioration in corneal stiffness.

Collagenase treatment gradually deteriorates the ultrastructure and the stiffness of the cornea. These changes are significantly higher than the changes obtained with amylase treatment. The deterioration of the ultrastructure includes reduction in both the diameter and the D-periodicity of collagen fibrils. Incubation with crude collagenase has a greater effect on corneal samples than purified collagenase. The disruption of collagen fibril morphology results in reduction of nano-scale stiffness.

## Figures and Tables

**Table 1:** A summary of the groups and treatment parameters.

Samples	Amylase Group	Crude Collagenase Group	Purified Collagenase Group
**No. of Porcine Eyes**	6	6	6
**No. of Sections**	66	66	66
**Incubation time (min)**	40	15	15
**Enzyme concentrations (mg/mL)**	0.2, 0.4, 0.6, 0.8, 1, 1.2, 1.4, 1.6, 1.8, and 2	0.05, 0.1, 0.15, and 0.2	0.05, 0.1, 0.15, and 0.2
**Washing solution**	PBS	NaDTA	NaDTA

## Data Availability

The data presented in this study are available on request from the corresponding author.
